# For whom and under what circumstances does email message batching work?

**DOI:** 10.1016/j.invent.2022.100494

**Published:** 2022-01-07

**Authors:** Indy Wijngaards, Florie R. Pronk, Martijn J. Burger

**Affiliations:** a Erasmus Happiness Economics Research Organization, Erasmus University Rotterdam, the Netherlands; b Erasmus School of Health & Policy Management, Erasmus University Rotterdam, the Netherlands; c Department of Organization, Open University of the Netherlands, the Netherlands

**Keywords:** Email batching, Interruptions, Well-being at work, Organizational intervention, HCI

## Abstract

Email plays an essential role in organizational communication but can also serve as pertinent source of work interruption and an impediment to well-being. Scholars have proposed email batching, processing emails only at certain times of the day, as a strategy to mitigate the negative consequences of email at work. As empirical evidence is mixed and applications in natural organizational contexts are lacking, we used survey data collected during a quasi-experimental top-down intervention in a Dutch financial services organization to investigate for whom and under what circumstances email batching is effective for reducing email interruptions and ameliorating well-being. We found that participants in the intervention group encountered less email interruptions than participants in the control group. Moreover, email batching reduced emotional exhaustion captured right after the intervention ended, especially for workers dealing with high email volumes and workers believing that instantaneous response was not expected in their organization. The effects of email batching wore off after two weeks and no significant effects on work engagement were found. We conclude that email batching should not be regarded as panacea for enhancing well-being and should only encouraged if it fits with workers' job tasks and organizational expectations regarding email response times more generally.

## Highlights

• We tested the effectiveness of an email batching intervention using a between-group quasi-experimental design.
• Email batching was negatively related to email interruptions and emotional exhaustion but was unrelated to work engagement.
• Email batching's effect on emotional exhaustion wore off after two weeks.
• Email batching especially reduces emotional exhaustion when worker deals with high email volumes
• Email batching especially reduces emotional exhaustion when organizations do not expect fast email response times.

## 1 Introduction

“The trick is to turn it off and only check occasionally and people do not expect immediate answers. If it is urgent, they can phone me.”

Pignata et al. (2015)

Email continues to be the most ubiquitous medium for organizational communication (Barley et al., 2011; Ragan, 2020; Rosen et al., 2019; Taylor et al., 2008). A recent survey among US workers in administrative or management roles suggested that, on average, workers spend over 3 h per day on the exchange of work-related email (Adobe, 2019). Another study revealed that 75% of US workers working in small to medium-sized businesses replies to email within 1 h, and 53% expects colleagues to do the same (Kelleher, 2013).

The use of email in the workplace has promises and pitfalls (Wajcman and Rose, 2011). Email is functional for organizational communication, building good interpersonal relationships, and promoting adequate job performance (Lowry et al., 2009; Mano and Mesch, 2010; Sheer and Rice, 2017; Ten Brummelhuis et al., 2012; Wajcman and Rose, 2011). For example, Wajcman and Rose (2011) demonstrated that for many workers high connectivity is pivotal for staying informed on task statuses and new developments, and getting work done. At the same time, for many, receiving, processing and answering online messages serve as most prominent sources of interruption that can significantly thwart their well-being (Fonner and Roloff, 2012; Puranik et al., 2020; Taylor et al., 2008). Several studies have shown that frequent email interruptions and high connectivity can instigate work overload, time pressure, job dissatisfaction, work disengagement, stress and feelings like anger and sadness (Barley et al., 2011; Derks et al., 2021; Jerejian et al., 2013; Mano and Mesch, 2010; Sonnentag et al., 2018; Ten Brummelhuis et al., 2012). Hence, it is not email per se that poses a problem to worker well-being and performance, but rather the continuous influx of work interruptions it brings when workers do not restrict the frequency of email interaction.

To address the interruption caused by emails at work, scholars have investigated the effectiveness of email batching – processing emails only at certain times of the day (e.g., Kushlev and Dunn, 2015; Robbins, 2004; Mark et al., 2012; Dabbish and Kraut, 2006; Mark et al., 2016). They consider email batching a useful email management strategy because it could reduce the total number of daily email interruptions and consequent occurrences of task switching, which in turn alleviates workers' overall cognitive strain (Kushlev and Dunn, 2015). This upkeep of cognitive effort and the continuance of the workflow allows workers to make adequate goal progress and keep exhaustion and negative emotions at bay (Puranik et al., 2020).

At the same time, empirical studies on the effectiveness of email batching have yielded mixed results (Mark et al., 2016 see the discussion in the next subsection). The potential reasons for this inconclusive evidence are manifold. It could be that the positive interruption-reducing effects of email batching are cancelled out by the distress that not attending an overflowing inbox brings (Dabbish and Kraut, 2006) and the discomfort associated to disrupting habits (Gardner, 2015; Wood et al., 2005). Alternatively, it may be that the relevance of email batching depends on individual differences (Akbar et al., 2019), the importance of emailing to get work done (Mark et al., 2016), or the organizational expectations regarding responsiveness (Barley et al., 2011; Reinke and Chamorro-Premuzic, 2014). Therefore, we used data collected during a top-down HR intervention within a Dutch financial services organization in a quasi-experiment to investigate *for whom* and *under what circumstances* email batching is effective for reducing email interruptions and supporting well-being. In the subsection below, we first elaborate on theoretical underpinnings of email batching and give an overview of relevant empirical evidence.

### 1.1 Theoretical underpinnings and empirical evidence

There are several psychological theories that can explain why interruption-induced task switching is associated with higher cognitive load and depleting available cognitive resources (Kushlev and Dunn, 2015; Puranik et al., 2020). Research building upon the *memory for goals theory* (Altmann and Trafton, 2002; Trafton et al., 2003) holds that the goals of the suspended interrupted task decay from memory during an interruption and cause resumption and completion times of the interrupted task to be higher and performance to be lower (Altmann et al., 2014; Altmann and Trafton, 2007). According to the *control theory of self-regulation* (Carver and Scheier, 1990) and *action regulation theory* (Hacker, 2003), workers will as a result exhaust their reservoirs of self-regulatory resources – cognitive resources that govern self-regulation, “the modification of habitual, natural, or dominant response” (Hamilton et al., 2011, p. 14) – to reorganize their work sequences and set things straight. The *time-based resource sharing model of attention* (Barrouillet et al., 2004) explains that even the very act of switching between tasks requires cognitive effort (Liefooghe et al., 2008). Finally, the *load theory of attention* (Lavie, 2010) argues that a high cognitive load and deleted reservoirs of cognitive resources could make people even more prone to distractive stimuli and motivate them to task switching, resulting in a spiral of cognitive resource loss (Lavie et al., 2004; Lavie and De Fockert, 2005; Leroy, 2009).

Email interruptions not only drain (cognitive) energy, they can also negatively affect well-being. As most workers perceive the continuous engagement in a certain work task as a pleasurable, behavioral momentum (e.g., flow, work absorption, Bakker et al., 2004; Csikszentmihalyi and Csikszentmihalyi, 1988), an interruption will be regarded as an unwelcome event and trigger a negative emotional response. Furthermore, following *affective events theory* (Weiss and Cropanzano, 1996), email interruptions are likely perceived as incompatible with goal progress and goal attainment and, for this reason, thwart well-being (Puranik et al., 2020). In a daily diary study, Sonnentag et al. (2018) showed that interruptions due to emailing at work led to more time pressure, which in turn elicited negative affective responses. Similarly, Baethge and Rigotti (2013) showed that hindered goal progress due to work interruptions can result in increased time pressure and feelings of irritation. Notably, negative affective responses may further fuel cognitive resource loss. The *conversation of resources theory* (Hobfoll, 1989, Hobfoll, 2001) and *ego-depletion theory* (Baumeister et al., 1998; Baumeister and Vohs, 2007) predict that workers are forced to use self-regulatory resources to suppress the negative affective responses caused by interruptions in the workplace (Lin et al., 2013).

In sum, it can be argued that email interruptions lead to the depletion of cognitive resources and trigger negative affective response. Email batching has the potential to reduce these interruptions, herewith being more beneficial for well-being than checking online messages continuously. As mentioned earlier, the support for this hypothesis is mixed. In a within-subjects field experiment, Bradley et al. (2013) showed that checking email once a day induces less stress than checking email continuously as usual. Using a similar research design, Kushlev and Dunn (2015) found that participants experience less stress on days that they checked email three times a day than when they had no limits. However, the effect on other well-being outcomes was limited. Blank et al. (2020) lab experimentally showed that participants that were exposed to continual email interruptions experienced more negative emotions during task completion than participants that received emails in a single batch. In contrast, in a correlational study, Dabbish and Kraut (2006) showed that restricting the moments of checking email, rather than checking email when a message came in, was associated with email overload. Drawing upon computer logs, biosensors and daily surveys of 40 knowledge workers, Mark et al. (2016) documented a non-significant correlation between email batching behaviors and stress. Using similar kinds of data, Bradley et al. (2013) showed that only 12% of respondents handled email in batches, and hypothesized that the unpopularity is likely due to workers perception that email batching has limited promise for stress prevention. In a lab study, Akbar et al. (2019) showed that email batching alleviates stress for emotionally stable participants and aggravates stress for those scoring higher on the neuroticism spectrum. Follow-up research by Akbar et al. (2021) suggested that email batching might not be a relevant email management strategy for all types of professionals.

### 1.2 Present research

In this study, we make use of data collected in a between-subjects quasi-experiment within a Dutch financial services organization to test the hypothesis that checking email during three batches a day (i.e., intervention condition) leads to less email interruptions and better well-being than checking email continuously as usual (i.e., control condition).

Well-being was captured using two variables, emotional exhaustion and work engagement. We adopted this multi-dimensional approach, because email batching (Kushlev and Dunn, 2015; Mano and Mesch, 2010; Jerejian et al., 2013) and organizational interventions more generally may not affect different aspects of well-being to a similar degree (Briner and Walshe, 2015; Nielsen et al., 2010a; Wijngaards et al., 2021). We selected emotional exhaustion, “a state of depleted work-related emotional and motivational resources” (Halbesleben et al., 2013, p. 493) and the main constituent of burnout (Seidler et al., 2014), as research drawing upon resource-based theories have often treated it as an indicator of low energy and negative sentiment as a result of depleted self-regulatory resources (Hobfoll et al., 2018; e.g., Lam et al., 2017; Lin et al., 2013; Wheeler et al., 2013). We chose work engagement, “a positive, fulfilling, work-related state of mind that is characterized by vigor, dedication, and absorption” (Schaufeli et al., 2002, p. 74) and the antipode of burnout. Work engagement was selected, because email interruptions likely take a heavy toll on workers' energy resources (threatening vigor), may be demotivating due to their negative association with goal progress (threatening dedication) and could hamper prolonged captivation in the job (threatening absorption, Parke et al., 2018).

We extend experimental research on the topic of email batching in two ways. First and foremost, in contrast to participants in previous experimental research, participants in our study did not self-select into the email batching intervention. Instead, the treatment was delivered as a top-down HR intervention by the organization itself – planned, behavioral, theory-based actions aimed at improving worker health and well-being by transforming the design, organization and management of work (Nielsen et al., 2010b). This research design allows us to empirically verify the recent theoretical proposition that the effectiveness of online message batching depends on individual and contextual factors (Fitz et al., 2019; Kushlev and Dunn, 2015) and examine whether email batching is also effective in a real-world setting. We considered the role of preference for multi-tasking, the intensity of email batching intervention, email volume and organizational expectations for email response times as relevant factors. In addition, next to effects tests, we evaluated the intervention process, e.g., satisfaction about the intervention, intent to use email batching in the future and suggestions for improvements (Nielsen et al., 2010a; Randall et al., 2009). Second, we investigated whether the intervention effects were sustainable over time by estimating well-being effects based on well-being data collected right after the intervention ended and data from a follow-up survey two weeks later.

## 2 Material and methods

### 2.1 Procedure

The email batching intervention that is studied is part of a HR program of a regional branch of a Dutch financial services organization aiming to improve worker well-being. The authors were asked to (1) develop an intervention that could help workers deal with the struggles associated with remote working, (2) recommend questions for the survey evaluation of the intervention, and (3) analyze the data. The intervention was developed by the second and third author and was based on the Kushlev and Dunn's (2015) experiment. With support of the second author, the HR department further tailored the intervention to the organization context and needs and implemented it. The data collection was administrated through HR and a third party that conducts all worker well-being surveys within the organization. The HR department presented the intervention to the participants as an ‘email challenge’ instead of a (quasi)-experiment or intervention, because workers in the organization are exposed to ‘challenges’ regularly (e.g., a step challenge).

**Fig. 1:**
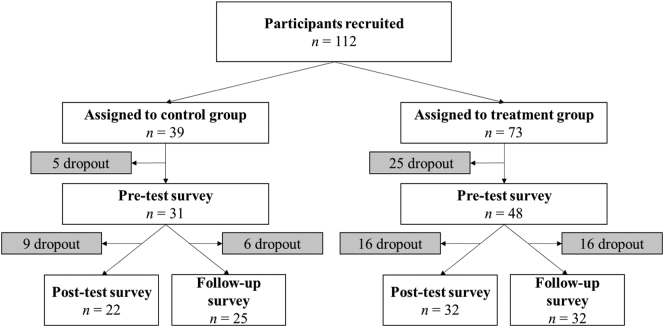
Participant flowchart. Notes. *n* = sample size.

Prior to the start of the intervention, the HR department assigned teams to ‘intervention’ and ‘control’ conditions based on geographic location, since a randomized experimental setup was impossible due to a risk of contamination. Consequently, as shown in the participant flowchart in Fig. 1, from the 112 selected workers, 39 nested in three teams were assigned to the control condition and 73 nested in four teams were assigned to the intervention condition. Within the intervention group, 39 participants were invited for an additional challenge. This challenge asked participants to also batch their instant messages (IM) three times per day. In the design of the experiment, we hypothesized that participants that only received an email batching intervention may compensate their unfulfilled need to check email by continuously checking their IM platforms. The more intensive intervention allowed us to control for this potential confounder in the analyses. The intervention period was one month.

The intervention group was introduced to the idea of email batching in an interactive, 1-hour (virtual) kick-off session, hosted by an HR officer and the second author from the organization. The managers of the participating teams and the regional director were also present. In the briefing, the HR officer and, in particular, the second author explained the reason behind this intervention and challenged how participants could alternatively manage their email notifications. Specifically, participants were explained how to change continuous email notifications to email notifications in batches on their computer and phone and were encouraged to (max 3) schedule blocks in their online agenda during which email could be answered and set up several reminders for the surveys. The case for the intervention was made by reporting on an earlier survey in the host company: A study among 446 workers in June 2020 showed that workers struggled to concentrate in their remote offices during the coronavirus pandemic and scored very high on the question “My work requires a lot of attention and concentration” (*M* = 4.08, *SD* = 0.71) that was answered on a five-point Likert scale (Bakker, 2014), with answer categories ranging from 1 (*completely disagree*) to 5 (*completely agree*). The sessions were recorded so that participants who were not able to attend were able to watch the session at their own convenience. Of the respondents who filled out both the pre-test and follow-up survey, 84% of participants attended the kick-off session in person, 9% did not but watched the recording and 7% did neither. It should be noted that participation in the email batching intervention was completely voluntary, but encouraged by the organization (e.g., participants had to alter their own settings for notifications and were free to check their email if they felt like it).

After the kick-off meeting, participants in the intervention group received three emails from the HR department: an email with the recording of the kick-off meeting and a summary of the most important insights, an invitation to participate in the pre-test survey, and a reminder for the pre-test survey. Participants were instructed to commence the intervention after they completed the pre-test survey. During the intervention, participants could contact HR and their respective managers for support. In the three weeks after the pre-test survey (thus during the intervention), participants received email invitations for intermediate weekly surveys. In the week after the intervention ended, participants received an invitation to the post-test survey. Two weeks after the post-test survey, participants were invited for a follow-up survey and received an email in case they did not complete the survey. Participants in the control group received an introductory email from HR that described that their organization wants to know more about the role of email and IM in the working lives of workers and that they were asked to provide this input. Like the participants in the intervention groups, participants in the control group received a pre-test survey, three intermediate surveys, a post-test survey and a follow-up survey. Once the intervention ended, it was up to the participant to decide whether they would continue to batch their email. In all surveys, respondents were asked for informed consent. Once all data was collected and analyzed, all participants and their managers received a debriefing on the study design and research findings.

### 2.2 Sample

From the total sample of 112 selected workers, 79 completed the pre-test survey (response rate = 71%). Of the participants that completed the pre-test survey, 53 completed the post-test survey and 57 completed the follow-up survey. A more detailed account by condition is provided in the participant flowchart in Fig. 1. We described the emailing behavior of the participants in the sample based on the pre-test survey data. The attrition analyses were also based the pre-test data. The demographics of the sample were described based on the follow-up survey, as it was in this survey that the demographic profile of participants was captured.

Of the 79 participants, 94% indicated that email is an important part of their job and 60% indicated that they found the exchanging of email a pleasant work task. In total, 30% received 25+ emails per day and 21% indicated that the daily number of emails results in stress. Seventy-one percent of participants indicated that the organization expects them to respond to emails quickly. The majority of the participants was male (68%) and aged 45 or older (70%). Participants worked in a variety of departments, including insurance and private banking. Most participants worked between 31 and 40 h per week (73%); only 5% had a managerial position. Because of the government rules to mitigate the spread of the coronavirus, all participants worked mostly from home.

As a substantial number of participants dropped from the post-test survey and follow-up survey, we followed Goodman and Blum's (1996) approach to test for systematic response differences by conducting two multiple logistic regression analyses: one with participation to the post-test survey as dependent variable and one with participation to the follow-up survey as dependent variable. We considered nine predictors in both regressions: assigned group, email volume, importance of email, the pleasantness of emailing at work, the stressfulness of high email volume, organizational expectations regarding email response times, preference for multi-tasking, work engagement and emotional exhaustion. Attrition analyses indicated that organizational expectations regarding email response times (*B* = 0.09, *p* = .048) and high email volume (*B* = 0.24, *p* = .046) were positively related to drop-out in the post-test survey and the stressfulness of high email volume was negatively associated to dropout (*B* = −0.13, *p* = .011). Dropout in the follow-up survey was only significantly related to the stressfulness of high email volume (*B* = −0.11, *p* = .025). It thus seems that there is a degree of self-selection in the current quasi-experiment: participants experiencing a high email volume and participants who feel that the organization does not support delayed email response times are underrepresented in this study. These findings are not surprising, as workers with a high workload are more prone to non-response than workers with a low workload (Rogelberg et al., 2003). In the current context, email volume and perceived norms for fast response times may be an indicator of high (perceived) workload. Participants who experience stress from their email volume are overrepresented. This finding can be explained by the fact that participants that are not in need of a well-being intervention will drop out of an intervention quicker and neglect survey invitations about the intervention (Lyubomirsky et al., 2011; Nielsen and Noblet, 2018). On a more general note, it is plausible that a proportion of the participants dropped out, because of the turbulence that the coronavirus pandemic caused in their professional and private lives (e.g., sickness absence, poor internet connection).

### 2.3 Measures

All measures used in this study were based on self-reports. All items were in Dutch. Because of demands from the organization, shortened scales and single-item measures were included in the survey. The measures were summarized in Table 1 (category, construct, schedule, participants, source, number of items, items and response categories). The internal consistency of the multi-item scales was considered satisfactory, as Cronbach's α values exceeded 0.8.

**Table 1:** Measures and descriptive statistics for study outcomes.

Category	Construct	Schedule	Participants	Source	# items	Item details	Response scale	α
Manipulation check	Estimated change in email checking behavior	Intermediate surveys, Post-test survey	Intervention	–	1	“Did you succeed in checking your work-related email maximally three times a day last week?”	1 – Never 7 - Always	–
		Follow-up survey	Intervention	–	1	“Did you succeed in limiting the frequency of checking work-related email to maximally three times a day during the entirety of the email challenge?”	1 – Never 7 - Always	–
Interruptions	Daily email interruptions	Intermediate surveys	Intervention and control	Ten Brummelhuis et al. (2012) and Sonnentag et al. (2018)	3	“Today, incoming work-related emails kept me from doing my job.” “Today, work-related emails have reached me at inconvenient moments.” “Today, work-related emails disturbed me in doing my work.”	1 – Never 7 - Always	0.87–0.90
Well-being	Emotional exhaustion	Pre-test survey, post-test survey, follow-up survey	Intervention and control	Maslach et al. (1986) and Schaufeli and Van Dierendonck (2001)	4	“I feel emotionally drained from my work.” “I feel used up at the end of the workday” “I feel fatigued when I get up in the morning and have to face another day on the job.” “A full day of work feels like a heavy burden for me.”	1 – Never 7 - Always	0.89–0.94
	Work engagement	Pre-test survey, post-test survey, follow-up survey	Intervention and control	Schaufeli et al., 2006, Schaufeli et al., 2019	3	“At my job, I feel bursting with energy.” “I am enthusiastic about my job.” “I am immersed in my job”.	1 – Never 7 - Always	0.83–0.86
Moderating variables	Preference for multitasking	Pre-test survey, post-test survey	Intervention and control	Poposki and Oswald (2010)	1	“If I had to choose between focusing on one task or multi-tasking, I would rather focus on just one task”.	1 – Never 7 - Always	–
	Email volume	Pre-test survey, post-test survey	Intervention and control	–	1	“How many work-related emails do you receive daily, on average?”	0–24 emails 25 or more emails	–
	Organizational expectations for email response times	Pre-test survey, post-test survey	Intervention and control	Day et al. (2012)	1	“In my organization, it is expected that I quickly respond on emails.”	1 – Never 7 - Always	
Variables for additional analyses	Satisfaction with intervention	Follow-up survey	Intervention	–	5	“How satisfied are you about the email challenge regarding the following aspects? - the challenge guidelines; - the challenge's effect for yourself; - the degree to which the challenge can be implemented in the daily work practice; - the usefulness of the challenge for your own work; - the communication surrounding the challenge”	1 – Very dissatisfied 7 - Very satisfied	–
	Reasons for not following intervention guidelines	Follow-up survey	Intervention	–	1	“What was the main reason for not being able to batch email to three times a day? Multiple options are possible.”	Own temptation to check; Client-related matters; Notifications; Colleagues; Others, namely …	–
	Motivation to batch email in the future	Follow-up survey	Intervention	–	1	“Do you feel motivated to regulate your emailing behavior in the future? Give a score between 1 and 10, where 1 stands for ‘not at all’ and 10 for ‘very much’.”	1 – Not at all 7 – Very much	–
	Aspects of email batching to sustain in the future	Follow-up survey	Intervention	–	1	“Which elements from the email challenge do you want to sustain in the future? Multiple options are possible.”	Switching email notifications off; Exchanging emails in batches Reducing overall email time; Making agreements with the team on emailing behavior; Beginning the day without email; Other, namely …	–
	Support in the future	Follow-up survey	Intervention	–	1	“Do you need support from [the organization] with regard to email management strategies?”	Yes; No	
	Suggestions for email batching interventions	Follow-up survey	Intervention	–	1	“According to you, what does it take to make this challenge to a success? This will help us with the design of interventions on happiness at work in the future.”	Open-text box	

Notes. IM = instant messaging, α = Range of Cronbach's α values across survey waves.

The manipulation check was based on several single-item measures of the successfulness of the manipulation. In specific, the actual change and estimated change in email checking behavior were measured. Three outcome measures were included. Daily email interruptions were measured using adapted 3-item scales (Sonnentag et al., 2018; Ten Brummelhuis et al., 2012). Work engagement was measured using the Dutch 3-item Utrecht Work Engagement Scale (Schaufeli et al., 2006, Schaufeli et al., 2019). Emotional exhaustion was captured using the Dutch 4-item Utrecht Burnout Scale (Schaufeli and Van Dierendonck, 2001; cf. Maslach et al., 1986). The pre-test survey and follow-up survey included single-item measures of preference for multi-tasking, email volume and organizational norms about email response times. We used administrative data to classify participants in the high-intensity and low-intensity intervention groups. As a general rule, the data from the pre-test survey was used. In case participants did not fill out the pre-test measure, data from the follow-up survey was used. The follow-up survey contained single-item measures on satisfaction with particular aspects of the intervention, reasons for not following intervention guidelines, motivation to batch email in the future, aspects of email batching to be sustained in the future and suggestions for email batching interventions.

### 2.4 Analytical strategy

We did a manipulation check by asking participants for their perceived following of the manipulation guidelines and reporting the descriptive statistics. The omnibus tests regarding the main effect of email batching on email interruptions, emotional exhaustion and work engagement were based on analysis of variance (ANOVA) tests. For the ANOVA test, effect sizes were computed based on partial eta squared (η^2^_*p*_); for the *t*-tests, they were computed based on Cohen's *d*. In the multivariate analyses, four dependent variables were considered. For both emotional exhaustion and work engagement, we considered two difference measures: one based on the difference between the pre-test survey and the post-test survey and the other based on the pre-test survey and the follow-up survey. The use of gain scores is justified by both the quasi-experimental setup and the use of a moderation analysis (Cribbie and Jamieson, 2000), as well as the main research question at hand: how do the experimental and the control groups differ with regard to changes in emotional exhaustion and work engagement (Fitzmaurice et al., 2012). We build models in steps. We started with estimating the main effect of email batching. Then, we estimated four models, each containing the main effect of email batching and one of the four moderators. Finally, we estimated a full model that included all variables. We considered age, gender, hours and department as control variables in the regression, but decided to refrain from presenting these results, because no control variables were statistically significant and including nonsignificant control variables unnecessarily reduces degrees of freedom (Bernerth et al., 2018). After the moderation analyses, we used data from the follow-up survey to contextualize the omnibus analyses and multivariate analyses. A *p*-value of .05 was considered statistically significant in the analyses.

## 3 Results

### 3.1 Manipulation checks

The mean scores on the item asking participants about the extent they were able to follow the intervention guidelines prompted in the intermediate surveys, scored on a 1–7 Likert scale, was 3.23 (*SD* = 1.61). Upon examination of the mean scores per survey wave, we found a downward trend: 3.38 (*SD* = 1.91) in the first intermediate survey, 3.26 (SD = 1.65) in the second, 3.12 (*SD* = 1.39) in the third and 3.06 (*SD* = 1.39) in the post-test survey. The follow-up survey question about the success in limiting the frequency of checking email behaviors three times a day over the entire course of the email challenge painted a somewhat more positive picture, with an average score of 4.53 (*SD* = 0.75). In summary, it can be concluded that, even though on a weekly basis participant did not feel that they were not able to fully comply to the intervention guidelines, the guidelines were generally followed over the course of intervention.

### 3.2 Omnibus effects

We found a marginal effect of batching on email interruptions. The mean score on the interruption index in the control group was 3.38 (*SD* = 0.75) and the mean in the intervention group was 2.90 (*SD* = 1.19), *t*_36.20_ = −1.82, *p* = .077, *d* = 0.51. Concerning our well-being outcomes, we found a significant negative omnibus effect of email batching on emotional exhaustion measured in the post-test survey (*F*_1,130_ = 9.04, *p* = .003, η^2^_*p*_ = 0.06, 95% CI = [0.01, 1.00]) and follow-up survey (*F*_1,134_ = 7.55, *p* = .007, η^2^_*p*_ = 0.05, 95% CI = [0.01, 1.00]). We found no support for the relationship between the intervention and work engagement, as indicated by nonsignificant omnibus in the post-test (*F*_1, 130_ = 0.14, *p* = .709, η^2^_*p*_ = 0.00, 95% CI = [0.00, 1.00]) and follow-up survey (*F*_1, 134_ = 0.33, *p* = .569, η^2^_*p*_ = 0.00, 95% CI = [0.00, 1.00]).

### 3.3 Multivariate analyses

In line with the results from the ANOVA tests and as exhibited in Table 2, email batching had a significant effect on the difference between emotional exhaustion measured in the pre-test survey and the post-test survey (Model 1). Moderation analyses showed that intervention intensity and preference for multi-tasking did not affect this relationship (Model 2 and 3). The analyses provided evidence for the moderating role of email volume and organizational expectations regarding email response times (Model 4 and 5, respectively). Specifically, for participants with a high email volume (receiving 25+ emails per day), email batching was more effective in lowering emotional exhaustion than it was for participants receiving little emails every day. For participants believing that their organization expects them to reply to emails quickly, the exhaustion-diminishing effects of email batching were less profound than for participants that believe the opposite. A model that includes all variables suggests that email volume and organizational expectations regarding email response times are robust moderators (Model 6).

**Table 2:** Regressions predicting the difference between emotional exhaustion in post-test survey and pre-test survey (*n* = 53).

	Model 1	Model 2	Model 3	Model 4	Model 5	Model 6
Email batching^a^	−0.29† (0.16)	−0.61 (0.45)	−0.23 (0.21)	−0.12 (0.17)	−1.61⁎⁎ (0.58)	−1.24† (0.70)
Preference for multi-tasking		−0.06 (0.07)				−0.02 (0.07)
Email volume^b^				1.04⁎⁎ (0.32)		0.92⁎⁎ (0.34)
Organizational expectations for email response times					−0.19⁎ (0.09)	−0.13 (0.09)
Email batching^a^ × preference for multi-tasking		0.07 (0.10)				0.03 (0.09)
Email batching^a^ × intervention intensity^c^			−0.09 (0.21)			−0.08 (0.20)
Email batching^a^ × email volume^b^				−1.11⁎⁎ (0.38)		−0.98⁎ (0.40)
Email batching^a^ × organizational expectations for email response times					0.28⁎ (0.12)	0.21† (0.12)
*R*^2^	0.06	0.07	0.07	0.23	0.16	0.29

Note. *R* = explained variance.

† *p* < .10.

⁎ *p* < .05.

⁎⁎ *p* < .01.

a 0 = “Control”, 1 = “Email batching intervention”.

b 0 = “less than 25 emails per day”, 1 = “25+ per day”.

c 0 = “Email batching intervention”, 1 = “Email and instant messaging batching intervention”.

As shown in Table 3, the results from regression analyses based on the difference between the pre-test survey and the follow-up survey did not reveal a significant effect of email batching on emotional exhaustion (*B* = −0.11, *p* = .500). A comparison of this effect size with the effect size of Model 1 based on the post-test survey data (*B* = −0.29) suggests that the effects of email batching wear off quickly. Additionally, in these analyses, no interaction terms reached statistical significance. This result diverges with the significant omnibus effect detected in the ANOVA tests. We expect that this discrepancy is caused by the fact that, in contrast to the regression analysis, an ANOVA test does not consider the baseline level of the independent variable. In addition, the regression analyses were performed on a smaller dataset (*n* = 57 vs. *n* = 136) and therefore may have lacked statistical power.

**Table 3:** Regressions predicting the difference between emotional exhaustion in follow-up survey and pre-test survey (*n* = 57).

	Model 1	Model 2	Model 3	Model 4	Model 5	Model 6
Email batching^a^	−0.11 (0.16)	−0.30 (0.47)	−0.03 (0.21)	−0.09 (0.19)	−0.11 (0.63)	0.70 (0.77)
Preference for multi-tasking		−0.03 (0.08)				−0.03 (0.08)
Email volume^b^				0.19 (0.33)		0.17 (0.33)
Organizational expectations for email response times					−0.01 (0.10)	0.02 (0.10)
Email batching^a^ × preference for multi-tasking		0.10 (0.10)				−0.12 (0.10)
Email batching^a^ × intervention intensity^c^			−0.16 (0.21)			−0.25 (0.23)
Email batching^a^ × email volume^b^				−0.15 (0.40)		−0.07 (0.40)
Email batching^a^ × organizational expectations for email response times					−0.04 (0.13)	−0.03 (0.13)
*R*^2^	0.01	0.09	0.02	0.01	0.11	0.12

Note. *R* = explained variance.

a 0 = “Control”, 1 = “Email batching intervention”.

b 0 = “less than 25 emails per day”, 1 = “25+ per day”.

c 0 = “Email batching intervention”, 1 = “Email and instant messaging batching intervention”.

Regression analyses confirmed the nonsignificant relationship between email batching and work engagement found in the ANOVA tests, as shown in Table 4, Table 5. The analyses did not reveal any significant moderators, except for email batching's interaction with the intensity of the treatment.

**Table 4:** Regressions predicting the difference between work engagement in post-test survey and pre-test survey (*n* = 53).

	Model 1	Model 2	Model 3	Model 4	Model 5	Model 6
Email batching^a^	0.04 (0.16)	0.49 (0.45)	0.14 (0.21)	−0.03 (0.18)	−0.37 (0.61)	−0.09 (0.76)
Preference for multi-tasking		0.04 (0.07)				0.04 (0.07)
Email volume^b^				−0.51 (0.35)		−0.59 (0.36)
Organizational expectations for email response times					−0.06 (0.09)	−0.11 (0.10)
Email batching^a^ × preference for multi-tasking		−0.10 (0.09)				−0.11 (0.10)
Email batching^a^ × intervention intensity^c^			−0.14 (0.21)			−0.16 (0.22)
Email batching^a^ × email volume^b^				0.53 (0.41)		0.58 (0.42)
Email batching^a^ × organizational expectations for email response times					0.09 (0.12)	0.14 (0.13)
*R*^2^	0.00	0.03	0.01	0.04	0.01	0.11

Note. *R* = explained variance.

a 0 = “Control”, 1 = “Email batching intervention”.

b 0 = “less than 25 emails per day”, 1 = “25+ per day”.

c 0 = “Email batching intervention”, 1 = “Email and instant messaging batching intervention”.

**Table 5:** Regressions predicting the difference between work engagement in follow-up survey and pre-test survey (*n* = 57).

	Model 1	Model 2	Model 3	Model 4	Model 5	Model 6
Email batching^a^	−0.02 (0.14)	0.76† (0.43)	−0.30 (0.18)	−0.05 (0.16)	−0.42 (0.57)	−0.27 (0.64)
Preference for multi-tasking		0.11† (0.07)				0.11 (0.07)
Email volume^b^				−0.65⁎ (0.28)		−0.60⁎ (0.28)
Organizational expectations for email response times					−0.07 (0.09)	−0.10 (0.08)
Email batching^a^ × preference for multi-tasking		−0.17† (0.09)				−0.13 (0.09)
Email batching^a^ × intervention intensity^c^			0.45⁎ (0.19)			0.42⁎ (0.19)
Email batching^a^ × email volume^b^				0.49 (0.33)		0.52 (0.33)
Email batching^a^ × organizational expectations for email response times					0.08 (0.11)	0.11 (0.10)
*R*^2^	0.00	0.07	0.10	0.10	0.01	0.25

Note. *R* = explained variance.

† *p* < .10.

⁎ *p* < .05.

a 0 = “Control”, 1 = “Email batching intervention”.

b 0 = “less than 25 emails per day”, 1 = “25+ per day”.

c 0 = “Email batching intervention”, 1 = “Email and instant messaging batching intervention”.

### 3.4 Additional analyses

#### 3.4.1 High satisfaction with the intervention

Overall, participants in the experimental group were satisfied with different aspects of the intervention (all measured on a 1–7 Likert scale). Nonetheless, substantial differences between the satisfaction scores were apparent. Participants were the most satisfied with the guidelines set in the intervention (*M* = 4.97, *SD* = 0.64), its usefulness for their jobs (*M* = 4.53, *SD* = 0.95) and the communication about the intervention (*M* = 5.44, *SD* = 0.80) and the least satisfied about the results of the intervention (*M* = 4.34, *SD* = 1.15) and how easy the intervention was to implement in their daily practice (*M* = 4.09, *SD* = 1.12).

#### 3.4.2 Client-related concerns as main reason for not following intervention guidelines

On the question why people failed to completely follow the intervention guidelines, participants most often mentioned client-related concerns (72%). Their own temptation (31%) and colleagues (28%) were also frequently mentioned. Of the ten participants that selected the ‘other reasons’ (31%), nine mentioned high dependence on email to do work effectively as a primary reason for why they did not follow the intervention guidelines. For example, “I obtained additional work tasks that come for 100% via email and instant messaging” and “My relations ask their questions via email and expect a prompt reply.” The one remaining participant reflected on the relevance of the intervention for his personal situation: “I don't experience pressure from incoming messages and work better if I know what comes in. I am perfectly able to find a balance and I know when to put my email aside.”

#### 3.4.3 Only a few aspects of the intervention were internalized once the intervention was ended

Of all the participants in the intervention group, 53% indicated that they continued email batching after the intervention was finished and 81% expressed an interest in retaining one or more aspects of the email batching intervention in their work. The most popular aspects were keeping email notifications off (66%) and batching email in blocks (56%). Less popular were reducing overall email time (34%), deciding on email-related norms within the team (22%) and starting the day without email (13%). Interestingly, 94% of participants indicated that no additional support regarding email management was desired in the future.

#### 3.4.4 Suggestions for improvements

Of the eighteen participants that offered concrete suggestions, eight commented to the impetus of considering job tasks when implementing email batching, e.g., “That you want to try it [email batching] yourself, especially if you are burdened by email (greater necessity)”, “[The effectiveness of email batching] is very dependent on your function and how client-oriented it is” and “It [email batching] has to align with the kind of work I do”. Six participants emphasized the importance of aligning organizational expectations regarding internal communication to the intervention guidelines, e.g., “Broader policy about approach and necessity of internal communication [is needed]. A multitude of messages does not mean that much information is shared”, “Clarity about the universality of the [new] way of working [is needed], so that no misunderstandings emerge about availability” and “Making arrangements within the team, for example, that one doesn't have to answer immediately”. Two participants reflected on the vitality of effective delivery of an email batching intervention: “You will get commitment of the participants by keeping it [the email batching intervention] simple. I think that you were successful in that regard.” and “I feel that the intervention was implemented well and the weekly email with questions were a reminder of the [email batching] challenge. If I wouldn't have received a reminder, the challenge wouldn't have been adopted as well, I think”. In sum, participants indicated that future email batching interventions should be offered to people whose job allows it, should be aligned with organizational norms surrounding internal communication, and should be delivered using multiple reminders.

## 4 Discussion

Building on several psychological theories and findings from earlier experiments of email batching, we evaluated a quasi-experimental field experiment to examine whether workers receiving an email batching intervention, as delivered as top-down HR intervention, experience higher well-being than workers in a control group that were asked to check as usual. More specifically, we investigated for whom and under what circumstances email batching is effective for reducing email interruptions, preventing emotional exhaustion and improving work engagement.

We found that most participants were able to adopt email batching in their daily practice during the experiment and it generally reduced email interruptions. Moreover, we documented a significant, negative association between email batching and emotional exhaustion and a nonsignificant association between email batching and work engagement. This finding is in line with research that suggests that email interruptions and strategies to reduce them have a stronger effect on negative well-being indicators than positive well-being indicators (Jerejian et al., 2013; Kushlev and Dunn, 2015; Mano and Mesch, 2010; Sonnentag et al., 2018; Ten Brummelhuis et al., 2012). Ten Brummelhuis et al. (2012) argued that virtual interruptions may be positively related to dedication and vigor at work due to increased perceptions of digital connectedness and negatively related to absorption at work due to a break of workflow, rendering the overall effect on work engagement nonsignificant. Sonnentag et al. (2018) showed that perceived interruptions predict positive affect via responsiveness to these messages.

Furthermore, we showed that the effects of the intervention on emotional exhaustion quickly wore off, although the majority of participants internalized one or more email batching behaviors after the intervention ended. This suggests that email batching is unlikely to lead to robust improvements in well-being if it is promoted as a temporary project. It is likely that for sustainable behavioral change and robust well-being improvements to occur, email batching should be integrated into the culture and core practices of an organization. Along with the finding that the overwhelming majority of participants did not have any desire for more support regarding email management once the intervention was ended, this result indicates that email batching should not be treated as magic bullet for ensuring high levels of worker well-being.

Finally, we demonstrated that the effects of email batching on emotional exhaustion varied across workers. First, workers that dealt with low email volumes reaped significantly less benefits from email batching than workers facing higher volumes. Receiving relatively little email will not only limit the amount of task switching and the pressure on workers' cognitive resources but will also lead to a small difference between checking email when it comes in as usual and checking email three times a day. This is in line with the earlier observation by Sonnentag et al. (2018), who found that work pressure mediates the relationship between email interruptions and negative affect. Second, email batching only seems to be effective if organizational norms do not dictate fast response time (Barber and Santuzzi, 2015; Barley et al., 2011; Brown et al., 2014; Day et al., 2012). Accordingly, it is unlikely that the implementation of email batching in an organizational unit will be successful if colleagues in other parts of the organization still expect a fast response time. Related to this point, additional analyses revealed that some jobs might not be suitable for email batching. We found that not only co-worker expectations but also client expectations play an important role in not completing the email batching challenge: when email batching interfered with achieving workers' goal to serve clients well, the email management strategy was perceived as a hindrance rather than a solution.

### 4.1 Limitations

There are several limitations to the present work. First, even though our focus on a single organizational context has allowed us to evaluate the practical feasibility of the email batching in an organization, it inherently limited the external validity of the findings (Landers and Behrend, 2015). For example, with the study taking place during the coronavirus pandemic, all study participants had to work from home and were completely reliant on virtual media to stay connected with colleagues and clients. Indeed, the Microsoft, 2021 Work Trend Index revealed that this transition from the office to work has globally led to a spike in email traffic (Microsoft, 2021). It is plausible that once participants are allowed to work from the office again, email volumes and email reliance diminish and email management strategies, such as email batching, become less relevant. Although participants in our sample worked in various departments and therefore seemed representative of a typical regional branch of a financial institution in the Netherlands, the effects may not generalize to other organizations in other industries and countries. For example, email batching may be more effective in organizations where the prompt satisfaction of *virtual* client needs is not central to work performance (e.g., healthcare workers, police officers and supermarket managers). As another example, with cross-cultural research showing that cultural differences exist in preferred email communication styles (Holtbrügge et al., 2013), it may be that the effectiveness of email batching is contingent on national culture. Against this background, we advise researchers to replicate the current study's findings in a diverse set of organizations.

Second, even though we adopted an inclusive approach toward operationalization of well-being and considered various moderators, we need more comprehensive research to help practitioners to make a case for email batching and understand the most important preconditions for effective implementation. For example, the construct of flow at work would have been an especially relevant well-being construct to consider, due to its close theoretical linkage with work interruptions (Baethge and Rigotti, 2013; Moneta, 2017). It would also be interesting to examine how email batching relates to dynamic well-being constructs, such as state work engagement (Breevaart et al., 2012) and state emotional exhaustion (Riedl and Thomas, 2019), and whether it has spillovers to well-being in the non-work domain (Becker et al., 2021; Ilies et al., 2007). More generally, it would be interesting to consider multimodal measurements of wellbeing, going beyond questionnaires. One can think here of including measurements of real-time emotions during email management, for example, through decoding video-taped facial expressions (Blank et al., 2020), analyzing heart rate variability (Mark et al., 2016) and using hair sample to derive cortisol levels (Ludwigs et al., 2020) to measure stress. An overview of innovative ways to measure worker well-being is provided within Wijngaards et al. (2021).

In addition, researchers are encouraged to consider performance-related outcomes, e.g., email response time (Gupta et al., 2011) and perceived productivity (Kushlev and Dunn, 2015), triangulate subjective and objective measures of email behaviors to control for recollection biases (Bargh, 2002; Collopy, 1996; Fitz et al., 2019) and include additional contextual moderators, such as telepressure (Barber and Santuzzi, 2015) and managers perceptions about the intervention (Nielsen, 2013; Randall et al., 2009). It would also be worthwhile for researchers to evaluate the role of individual personality traits for the effectiveness of an email batching intervention as well as the extent to which work-related email affects individuals' well-being differently when taking personality in account. While our measure of preference for multi-tasking turned out to be an insignificant moderator, there are several plausible personality trait interactions available, e.g., neuroticism (Akbar et al., 2019) and conscientiousness (Mark et al., 2016). Finally, future research may also benefit from investigating the effectiveness of the different components of an email batching intervention and alternative email management strategies, such as email filing and filtering (Dabbish and Kraut, 2006; Jerejian et al., 2013). Our additional analyses, for example, suggested that starting the day without email was perceived as infeasible by the majority of participants.

Third, the attrition in our sample is high and, provided that employees are likely to drop out sooner when email batching does not work for them, the eventual impact of email batching may even lower than reported. The disproportionate drop-out of employees with a high email volume is particularly worrisome in this regard. At the same time, the most plausible reason for this group to drop out are client-related concerns and existing organizational norms regarding response times. Hence, the high attrition rate merely underlines that email batching is not a sine qua non improving worker well-being and that the organizational context and culture are of pivotal importance for the implementability of this intervention.

### 4.2 Implications

This study corroborates findings that email handling strategies are more strongly related to negative, rather than positive, indicators of well-being (Jerejian et al., 2013; Kushlev and Dunn, 2015; Mano and Mesch, 2010) and empirically verifies the theoretical proposition that the effectiveness of online message batching may depend on individual and contextual factors (Fitz et al., 2019; Kushlev and Dunn, 2015). In this regard, our study corroborates with recent work by Akbar et al. (2021). The authors found that physicians spend small amounts of time on email exchange during scheduled patient meetings and attend clinically urgent emails in between patient meetings – a behavioral pattern that likely makes email batching an infeasible and potentially even undesirable email management strategy. More broadly, this study strengthens the case for focus on the question “what works for whom in which circumstances?” rather than the more general question “what works?” (Nielsen and Miraglia, 2017, p. 40; Nielsen et al., 2010a; Nielsen and Noblet, 2018) and links to the question why many organizational interventions derail (Karanika-Murray and Biron, 2015).

We suggest that organizations should not regard email batching as panacea for enhancing worker well-being as of yet. In case an organization wants to implement email batching, it is well-advised to foster a work climate where instantaneous email responses are discouraged prior to its introduction and only encourage workers to adopt this practice if it suits their jobs. Without the appropriate norms in the organization and without email being a considerable stressor at work, it is unlikely that an email batching intervention will change behaviors or improve worker well-being. Concretely, an organization may consider top-down communication of healthy email expectations, tailor the principles of email batching to the need of teams and develop an email protocol that helps workers to use email responsibly, e.g., putting email expectations in one's email signature and changing the default notification settings in email software (Giurge and Bohns, 2021). On a more general note, this study highlights that organizational interventions can derail or not completely succeed in various ways and the successfulness of interventions does not only depend on the content of the intervention, but also on the context it is implemented in. When an organization wants to make an enduring positive change, it needs to incorporate the intervention into daily practice.

## Declaration of competing interest

This work was supported by the 10.13039/501100003246Netherlands Organisation for Scientific Research (NWO) [652.001.003].

The authors have received commercial funding from the organization for data collection to evaluate this policy intervention initiated by the organization and have received consent to use the data for academic research. The funders had no role in the analysis, decision to publish, or preparation of the manuscript.
